# Persistent Bacteremia from* Pseudomonas aeruginosa* with* In Vitro* Resistance to the Novel Antibiotics Ceftolozane-Tazobactam and Ceftazidime-Avibactam

**DOI:** 10.1155/2016/1520404

**Published:** 2016-10-12

**Authors:** Louie Mar Gangcuangco, Patricia Clark, Cynthia Stewart, Goran Miljkovic, Zane K. Saul

**Affiliations:** ^1^Department of Internal Medicine, Yale New Haven Health-Bridgeport Hospital, Bridgeport, CT, USA; ^2^Department of Microbiology, Yale New Haven Health-Bridgeport Hospital, Bridgeport, CT, USA; ^3^Internal Medicine and Infectious Disease Associates P.C., Stratford, CT, USA

## Abstract

Ceftazidime-avibactam and ceftolozane-tazobactam are new antimicrobials with activity against multidrug-resistant* Pseudomonas aeruginosa*. We present the first case of persistent* P*.* aeruginosa* bacteremia with* in vitro* resistance to these novel antimicrobials. A 68-year-old man with newly diagnosed follicular lymphoma was admitted to the medical intensive care unit for sepsis and right lower extremity cellulitis. The patient was placed empirically on vancomycin and piperacillin-tazobactam. Blood cultures from Day 1 of hospitalization grew* P*.* aeruginosa* susceptible to piperacillin-tazobactam and cefepime identified using VITEK 2 (Biomerieux, Lenexa, KS). Repeat blood cultures from Day 5 grew* P*.* aeruginosa* resistant to all cephalosporins, as well as to meropenem by Day 10. Susceptibility testing performed by measuring minimum inhibitory concentration by *E*-test (Biomerieux, Lenexa, KS) revealed that blood cultures from Day 10 were resistant to ceftazidime-avibactam and ceftolozane-tazobactam. The Verigene Blood Culture-Gram-Negative (BC-GN) microarray-based assay (Nanosphere, Inc., Northbrook, IL) was used to investigate underlying resistance mechanism in the* P*.* aeruginosa* isolate but CTX-M, KPC, NDM, VIM, IMP, and OXA gene were not detected. This case report highlights the well-documented phenomenon of antimicrobial resistance development in* P*.* aeruginosa* even during the course of appropriate antibiotic therapy. In the era of increasing multidrug-resistant organisms, routine susceptibility testing of* P. aeruginosa* to ceftazidime-avibactam and ceftolozane-tazobactam is warranted. Emerging resistance mechanisms to these novel antibiotics need to be further investigated.

## 1. Introduction

Sepsis from* Pseudomonas aeruginosa* bacteremia may be fatal and necessitates prompt antimicrobial therapy. Newer antimicrobials have been developed to address the rise of multidrug-resistant* P. aeruginosa* [1]. Among these are ceftolozane-tazobactam, a combination of a fifth-generation cephalosporin and a *β*-lactamase inhibitor, and ceftazidime-avibactam, a combination of a third-generation cephalosporin and a non-*β*-lactam *β*-lactamase inhibitor [2]. We present the first case report of persistent* P. aeruginosa* bacteremia resistant to these novel antibiotics.

## 2. Case Presentation

A 68-year-old man presents to the Emergency Department for a 6-month history of worsening fatigue, anorexia, and weight loss. CT scan of the abdomen showed enlarged lymph nodes. Axillary lymph node biopsy showed follicular lymphoma with bone marrow involvement. The patient was started on chemotherapy with rituximab, etoposide, prednisolone, oncovin, cyclophosphamide, and hydroxyl-daunorubicin (R-EPOCH). Second cycle consisted of rituximab, cyclophosphamide, hydroxyl-daunorubicin, oncovin, and prednisone (R-CHOP). The patient had a prolonged hospital course (3 months) complicated by tumor lysis syndrome, febrile neutropenia (treated with aztreonam, cefepime, and anidulafungin), acute renal failure requiring hemodialysis, right lower extremity cellulitis treated with a 7-day course of intravenous vancomycin, and* Clostridium difficile* colitis treated with oral metronidazole.

On hospital discharge, outpatient chemotherapy consisted of bendamustine and rituximab. Two weeks later, the patient presented to the clinic for recurrence of right lower extremity cellulitis. Physical exam revealed erythema and induration of the right upper leg with extension to the groin and left medial thigh. One dose of intravenous ceftriaxone was administered and amoxicillin-clavulanate 500 mg three times a day was started. The patient presented 1 day later to the Emergency Department with increased shortness of breath, loose bowel movement, hypotension (80/40), tachycardia (129 beats/minute), temperature of 99.6°F, and oxygen saturation of 75% in room air. Empiric vancomycin and piperacillin-tazobactam were started and 6 liters of normal saline bolus led to improvement in blood pressure. Hospital course is summarized in Table 1.

Blood cultures from the day of admission grew* P. aeruginosa* (Table 2), identified using VITEK 2 (Biomerieux, Lenexa, KS). Blood cultures persistently grew* P. aeruginosa* initially susceptible to piperacillin-tazobactam and cefepime, with subsequent resistance to all cephalosporins and penicillins by Day 5 and resistance to meropenem by Day 10. Antimicrobial susceptibility testing to ceftolozane-tazobactam and ceftazidime-avibactam was performed for the* P. aeruginosa* isolate from Day 10 by measuring minimum inhibitory concentration using *E*-test (Biomerieux, Lenexa, KS). *E*-test showed 0 mm zone of inhibition for ceftazidime-avibactam (resistant) and 16 mm for ceftolozane-tazobactam (resistant).

The patient had an extensive work-up to find the source of* P. aeruginosa* bacteremia. Transesophageal echocardiogram did not show vegetation or endocarditis. Noncontrast CT scan of the chest, abdomen, and pelvis revealed bilateral pleural effusions but no abscess. Low platelet counts precluded thoracentesis. Urine cultures (Day 1, Day 10, and Day 15), as well as catheter tip culture of peripherally inserted central catheter line (PICC line), did not grow bacteria.

Antimicrobial coverage was adjusted appropriately based on blood culture susceptibility reports. Despite medical treatment, the patient developed progressive acidosis, respiratory distress, and hypotension. By Day 15, he required three vasopressors. A decision was made to shift the patient from full interventions to comfort measures only. He was extubated and expired on Day 16 of hospitalization.

To determine possible underlying resistance mechanisms in the* P. aeruginosa* isolate, the Verigene Blood Culture-Gram-Negative (BC-GN) microarray-based assay (Nanosphere, Inc., Northbrook, IL) was utilized following previously published methods [3]. In brief, we seeded a known negative blood culture bottle with the* P. aeruginosa* isolate using 100 mL of a 0.5 McFarland standard in normal saline. Blood culture bottles were placed on the BACTEC automated blood culture monitoring system (BD Diagnostics, Franklin Lakes, NJ, USA). Once they were flagged positive, we took four 1 mL aliquots and placed them in sterile tubes. The tubes were immediately placed in −70°F freezer before shipping on dry ice to Nanosphere, Inc. (Illinois, USA). The Verigene BC-GN detected* P. aeruginosa*. However, all resistance markers tested were negative (CTX-M, KPC, NDM, VIM, IMP, or OXA gene).

## 3. Discussion

We present the first documented case of persistent* P. aeruginosa* bacteremia resistant to the novel antimicrobials ceftazidime-avibactam and ceftolozane-tazobactam. A recent publication on a multidrug-resistant* P. aeruginosa* bacteremia was reported by Bremmer and colleagues, but the isolate was susceptible to ceftolozane-tazobactam [4]. In our current report, a rapid development of antibiotic resistance was observed despite appropriate antimicrobial therapy and, intriguingly, with resistance to ceftazidime-avibactam and ceftolozane-tazobactam, agents that have been only recently approved by the US Food and Drug Administration. This case raises the following important questions: (1) Must patients remain on double antimicrobial coverage for* P. aeruginosa* pending* repeat* blood cultures? (2) Should screening for resistance determinants be routinely performed for* P. aeruginosa*?

The rationale for double antibiotic coverage/combination therapy against suspected* P. aeruginosa* infection is to increase the chance that the patient receives an active agent awaiting final susceptibility results. Combination therapy also has a theoretical benefit in decreasing the emergence of resistance and may confer synergistic effect. Although the routine use of combination antimicrobials for* P. aeruginosa* remains controversial, there is evidence that a subset of patients who are at high risk for resistant strains (i.e., patients with neutropenia, burn, severe sepsis, or shock) may benefit from combination therapy [5]. Measuring peak and minimum inhibitory concentrations for ciprofloxacin and aminoglycosides was also shown to be associated with increased success/clinical cure in* P. aeruginosa* bacteremia [6].

In our current report, despite appropriate antimicrobial therapy and initial isolate susceptible to piperacillin-tazobactam, the patient continued to have bacteremia. There are two possible reasons for this: (1) the patient has multiple* P. aeruginosa* strains and the predominant phenotype from Day 1 was eradicated by piperacillin-tazobactam, with the carbapenem-resistant strains subsequently becoming the predominant phenotype in the repeat blood cultures or (2) the initial* P. aeruginosa* isolate developed resistance from mutation/acquisition of exogenous resistance determinants.

The ability of* P. aeruginosa* to develop resistance during antimicrobial therapy has been well-documented in literature and involves complex mechanisms, including chromosomally encoded AmpC cephalosporinase, outer membrane porin (OprD), and multidrug efflux pumps [7]. AmpC *β*-lactamases are chromosomally encoded cephalosporinases. AmpC enzymes may be induced and expressed at high levels by mutation. Overexpression of AmpC confers resistance to broad-spectrum cephalosporins, including ceftazidime [8]. Furthermore, structural modifications in AmpC may impact the ability of avibactam to protect ceftazidime from hydrolysis [9]. A study comparing wild-type and mutator* P. aeruginosa* strains showed that the development of high-level resistance to ceftolozane-tazobactam occurs in the presence of* P. aeruginosa* mutator strains and that multiple mutations result in overexpression and structural modifications of AmpC [10]. Resistance to carbapenems, on the other hand, can arise from a simple mutation in* P. aeruginosa* and one mechanism is through the loss of OprD, a carbapenem-specific porin [11].

We initially thought that the* P. aeruginosa* isolate from our current patient produces metallo-*β*-lactamase, which confers resistance to both cephalosporins and carbapenems [12]. However, it was surprising that the Verigene BC-GN did not detect any of the carbapenemases tested (KPC, NDM, VIM, IMP, or OXA gene). The resistance that we observed may be due to a mechanism not in the BC-GN panel, or the* P. aeruginosa* strain may have acquired resistance through other novel mechanisms. Unfortunately, our limited resources precluded us from testing the isolate for possible chromosomal resistance mechanisms at a reference laboratory.

Another limitation of our case report is the lack of testing of the initial isolate's susceptibility to ceftolozane-tazobactam and ceftazidime-avibactam from Day 1. Per our Microbiology lab protocol, positive blood culture samples are discarded 1 week after the Microbiology report has been finalized. When the patient's blood culture sample from Day 10 showed resistance to meropenem and the decision was made to test for ceftolozane-tazobactam and ceftazidime-avibactam, blood sample from Day 1 was no longer available. Hence, susceptibility testing was performed only on the most recent blood culture (Day 10).

Unlike the presence of extended-spectrum *β*-lactamase production in* Enterobacteriaceae*, the presence of resistance determinants in* P. aeruginosa* is not routinely tested in most hospitals. Whether testing for carbapenemases and other resistance determinants prior to initiation of antimicrobial treatment would impact mortality among patients with* P. aeruginosa* bacteremia remains to be elucidated. Continued antimicrobial susceptibility surveillance, development of cost-effective screening tests for antimicrobial resistance, and further studies on appropriate treatment strategies in persistent* P. aeruginosa* bacteremia are warranted.

## Figures and Tables

**Table 1:** Significant events in the patient's hospital course.

Hospital day	Significant event	*T*max (°F)	WBC (cells/mm^3^)	Antibiotics
1	Admission to the medical ICU, being started on norepinephrine for hypotension Blood cultures drawn Right radial A-line and left femoral central line inserted	103.0	4.61	Piperacillin-tazobactam 4.5 g IV every 8 hours Vancomycin IV^*∗*^
2	Noncontrast CT scan of abdomen and pelvis showed bilateral pleural effusion, moderate ascites, generalized anasarca, no abscess	100.7	11.16	
3	Day 1 blood cultures grew *P. aeruginosa* Stool *C. difficile* enzyme immune-assay positive^*∗∗*^	99.8	15.60	Cefepime 2 gm IV daily
4	Rising creatinine (1.8 mg/dL) Urinary catheter removed Femoral line removed PICC line inserted	98.3	17.07	
5	Hemoglobin decreased from 7.2 to 6.8 g/dL Platelets decreased from 27 to 16 × 10^3^/microliter 1 unit of packed RBC transfused Repeat blood cultures drawn	97.7	14.29	
6	Blood pressure stable off vasopressor, arterial line removed Repeat blood culture negative to date Transferred to general medical floor	97.8	6.38	
7	Repeat blood culture × 1, no growth Minimal bleeding from nares, platelets transfused	97.8	12.4	
8	Blood culture from Day 5 resistant to cefepime	98.2	11.93	Meropenem 1 g IV every 8 hours
9	1 unit of packed RBC transfused	99.0	9.68	
10	Transthoracic 2D ECHO showed possible valvular vegetations HIDA scan, negative Intubated for decreased respiratory rate and apnea, hypotension (75/50), altered mental status Repeat blood cultures were drawn Transferred back to the medical ICU	98.4	5.96	Meropenem 1 g IV every 8 hours + tobramycin^*∗*^
11	Hypotension despite fluid resuscitation Phenylephrine started	98.0	6.29	
12	Transesophageal echocardiogram did not reveal vegetations PICC line removed and sent for culture Stable respiratory status; patient extubated	98.3	4.35	
13	Worsening renal function and oliguria Blood culture from Day 10 grew *P. aeruginosa* resistant to meropenem; *E*-test performed for *P. aeruginosa* isolate from Day 10 showed resistance to ceftazidime-avibactam and ceftolozane-tazobactam	99.3	3.84	Tobramycin 1.7 mg/kg every 12 hours
14	Increasing tachypnea, tachycardia, lethargy	98.5	1.54	
15	Absolute neutrophil count dropped to 590 cells/mm^3^ Reintubated for respiratory distress Hypotension despite fluids; patient on three vasopressors (norepinephrine, phenylephrine, vasopressin) Repeat blood cultures drawn	98.5	2.53	Tobramycin 1.7 mg/kg every 12 hours Anidulafungin 200 mg IV Cefepime 2 gm IV daily Vancomycin IV
16	Family decided to change the patient's code status from full interventions to comfort measures only			
	He was extubated and expired			

^*∗*^Tobramycin and vancomycin doses were adjusted by pharmacy based on peak and trough blood levels.

^*∗∗*^Patient was on oral vancomycin empirically for *C. difficile *since Day 1 of hospitalization.

HIDA scan: hepatobiliary iminodiacetic acid scan; IV, intravenous; PICC: peripherally-inserted central catheter; and *T*max, maximum temperature.

**Table 2:** Antimicrobial susceptibility of *Pseudomonas aeruginosa* isolated from the blood.

Antimicrobial	Day 1		Day 5		Day 10^*∗*^		Day 15	
	MIC	Int	MIC	Int	MIC	Int	MIC	Int
Piperacillin-tazobactam	32	S	≥128	R	≥128	R	≥128	R
Cefepime	8	S	32	R	≥64	R	≥64	R
Aztreonam	16	I	16	I	≥64	R	≥64	R
Meropenem	4	S	4	S	≥16	R	≥16	R
Amikacin	16	S	16	S	16	S	16	S
Gentamicin	8	I	8	I	≥16	R	≥16	R
Tobramycin	≤1	S	≤1	S	≤1	S	≤1	S
Ciprofloxacin	≥4	R	≤4	R	≥4	R	≥4	R
Tigecycline	≥8	R	≤8	R	≥8	R	≥8	R

Int, interpretation; MIC, minimum inhibitory concentration; R, resistant; S, susceptible; and I, intermediate.

^*∗*^Antimicrobial susceptibility testing of *P. aeruginosa* against ceftolozane-tazobactam (C/T) and ceftazidime-avibactam (CZA) by *E*-test was performed for the *Pseudomonas* isolate from Day 10. *E*-test showed 0 mm zone of inhibition for CZA (resistant) and 16 mm for C/T (resistant). Cefepime was used as a surrogate for ceftazidime susceptibility.
